# Supplementary material for: MetaRibo-Seq measures translation in microbiomes
Source: Nat Commun. 2020 Jun 29;11:3268. doi: 10.1038/s41467-020-17081-z (PMC7324362; doi:10.1038/s41467-020-17081-z)
Supplement: Supplementary file 10 — Supplementary Data 7 [file 41467_2020_17081_MOESM10_ESM.zip › File2/Confidence_High_Taxonomy/157197_out.krona.html]

Javascript must be enabled to view this page.

members
magnitude
magnitudeUnassigned
count
unassigned
taxon
rank

157197\_out

6

4
superkingdom
2759

4751
4
kingdom

451864
4
subkingdom

SRS023914\_contig\_number\_15549
1

4890
1
phylum

147538
subphylum

SRS051031\_contig\_number\_contig-100\_500.195359
1

5204
phylum
2

subphylum
2
452284

class
2
1538075

2
order
162474

742845
family
2

55193
genus
2


SRS013687\_contig\_number\_17190SRS1041145\_contig\_number\_36319
species
2
2020962

2
2
superkingdom

1224
phylum
2

2
class
28211

204457
1
order

1
family
41297

13687
genus
1


SRS053356\_contig\_number\_contig-100\_353.134039
species
1
1390395

204455
order
1

1948890
species

SRS098571\_contig\_number\_64993
1
